# End of life care in UK care homes during the COVID-19 pandemic: a qualitative study

**DOI:** 10.1186/s12904-022-00979-4

**Published:** 2022-06-01

**Authors:** Kerry Hanna, Jacqueline Cannon, Mark Gabbay, Paul Marlow, Stephen Mason, Manoj Rajagopal, Justine Shenton, Hilary Tetlow, Clarissa Giebel

**Affiliations:** 1 School of Health Sciences, University of Liverpool, Liverpool, L69 3GB England; 2 Lewy Body Society, Wigan, UK; 3 Department of Primary Care & Mental Health, University of Liverpool, Liverpool, UK; 4 NIHR ARC NWC, Liverpool, UK; 5 Institute of Life Course and Medical Sciences, University of Liverpool, Liverpool, UK; 6 Lancashire and South Cumbria NHS Foundation Trust, Preston, UK; 7 Sefton Advocacy, Sefton, UK; 8 SURF Liverpool, Liverpool, UK

**Keywords:** Care sector, COVID-19 pandemic, Dementia, Older adults

## Abstract

**Purpose:**

To report the experiences of End of Life (EoL) care in UK care homes during the COVID-19 pandemic.

**Methods:**

UK care home staff and family carers of residents in care home took part in remote, semi-structured interviews from October to November 2020, with 20 participants followed-up in March 2021. Interviews were conducted via telephone or online platforms and qualitatively analysed using inductive thematic analysis.

**Results:**

Forty-two participants (26 family carers and 16 care home staff) were included in a wider qualitative study exploring the impact on dementia care homes during the pandemic. Of these, 11 family carers and 9 care home staff participated in a follow-up interview. Following descriptive thematic analysis, three central themes concerning EoL care during the pandemic specifically, were conceptualised and redefined through research team discussions: 1) Wasting or losing time; 2) Maintaining control, plans and routine; and 3) Coping with loss and lack of support. Lack of suitable, meaningful visits with people with dementia in care homes resulted in negative feelings of guilt and abandonment with both family carers and care home staff. Where families experienced positive EoL visits, these appeared to breach public health restrictions at that time.

**Conclusion:**

It is recommended that care homes receive clear guidance from the government offering equitable contact with relatives at EoL to all family members, to support their grieving and avoid subsequent negative impacts to emotional wellbeing.

**Supplementary Information:**

The online version contains supplementary material available at 10.1186/s12904-022-00979-4.

## Introduction

End of life (EoL) care is defined as care for people in the last year of life, with the aim of supporting people to live and die with dignity [1, 2]. Prior to the COVID-19 pandemic, there was a significant increase in the number of residents with dementia receiving EoL care in the care home setting, with care homes identified as the preferred place of care [3]. Hospitalisation, particularly in the last days and weeks of life, is considered stressful and is often avoidable [4]. During the pandemic, residents were frequently deemed unsuitable for hospital admission due to pressure on bed occupancy with the growing number of COVID-positive cases [5, 6]. In addition, further reports claim that residents and their families chose not to be admitted to hospitals and hospices where possible, due to pandemic-related visiting restrictions [7].

In the UK, care homes are not recognised as “frontline” NHS sites of care and so were not prioritised for intense support in the early stages of the pandemic, with many experiencing personal protection equipment (PPE) and staff shortages, which compromised care and negatively impacted care home residents through increased rates of COVID-19 infection and increased number of deaths [7, 8]. In addition, guidance on EoL care in care homes was found to be lacking internationally, and mostly focused on infection prevention, and not actively guiding care [9]. The World Health Organisation issued rapid guidance on maintaining essential health services during the early stages of the pandemic, however failed to specifically include both palliative and EoL care [7].

Visiting restrictions in hospitals and care homes prevented families from visiting their relatives during the pandemic [10], the emotional impact of which was far-reaching for both people living with dementia and family carers [11]. It is well established that meeting the needs of relatives when a family member is dying can help facilitate better psychological adjustment in their grief [12]. Early research into the experiences of bereaved families during COVID-19 has found many families were frustrated with the lack of communication from health and social care providers, which impeded their ability to appropriately prepare for the death of their relative [13]. Families who were able to visit their relative were twice as likely to report feeling adequately supported during the last days of life, compared to those unable to visit [13]. Subsequent qualitative research with a sample of the survey participants, corroborated findings of increased communication needs due to lack of visiting restrictions [14]. However, the impact that these restrictions have had on both the bereaved family carers and care home staff supporting residents specifically living with dementia, is still unknown.

Care home staff have also experienced negative emotional impacts during the time of the pandemic, including stress and burden from increased job demands, staff shortages, and caring for residents in isolation, in addition to the demoralising media coverage of care homes during that time [15]. Furthermore, a recent report from the UK House of Commons noted a rise in social care staff stress and burnout during the pandemic, recommending continued monitoring of COVID-19 on the adult social care workforce to support planning of the pandemic recovery [16], providing further rationale for the current study. It is possible that further impacts caused by caring for increased numbers of residents at EoL during the pandemic, will have further impacted staff’s emotional wellbeing, however, to date, the practicalities of delivering EoL care, and the subsequent impact that any changes to care delivery may have on the workforce and family carers of residents in care homes has not yet been reported in the current literature.

The aim of this research was, therefore, to examine the data from a wider qualitative study on the impact of COVID-19 restrictions on care homes with dementia residents, and to specifically report on the experiences of EoL care in UK care homes during the pandemic, to better inform future care provision and long-term recovery goals from the pandemic.

## Methods

### Participants and recruitment

The study is of a larger body of research, reporting the impact of the COVID-19 pandemic on residents’ families and care home staff [17]. The current sub-study analysed experiences of EoL care during the pandemic specifically. Eligibility for the study included care home staff who worked in a care home, or worked solely with care homes, as part of their clinical roles; family carers of a person living with dementia (PLWD) in a care home during the COVID-19 pandemic; and participants had to be aged ≥18. Third sector organisations advertised the study for recruitment and an existing network of dementia and ageing and social media were used to advertise the study. The study information was shared with organisations, and further information was further posted on social media. Interested participants then contacted the principal investigator via email and an approved participant information sheet was emailed to the participants, and re-read again prior to taking consent. Verbal informed consent was taken before the interview commenced, which was also audio-recorded, as per the approved ethical protocol.

### Data collection and setting

The COREQ checklist has been used to guide the reporting of this research [18]. Baseline qualitative data were collected between October and November 2020 from 42 participants [19]. Twenty of these (11 family carers and 9 care home staff), were purposely sampled for a follow-up interview in March 2021 when care homes had re-opened their doors to (restricted) visits, to identify how, if at all, participants’ experiences changed since the time of first interview, given the constantly changing circumstances and government guidance around care home visits at that time. For context, care homes were closed to all visits at the time of the baseline interviews, whilst the UK public health restrictions at the time of the follow-up interviews allowed a return to care home visits, albeit restricted with mandatory lateral flow testing and PPE [17, 20]. Earlier analysis of the follow-up interviews has been published elsewhere [17], outlining changed perceptions over time. However, a prominent, subsequent finding emerged from this work around EoL care at this time, which warranted further sub-analysis.

Ethical approval was granted to contact all participants from the baseline study via email, inviting them to take part in a follow-up interview. The participants who responded were invited to take part, ensuring a balanced representation of both care workers and family carers. Recruitment ceased when data saturation was observed. Eleven participants did not respond to the email invite, and no participant later refused to participate or dropped-out of the study. Semi-structured interviews were conducted online (zoom) or via telephone, with the use of a topic guide (see Additional file 1). Participants were initially asked demographic background questions, and staff were then asked to discuss changes to their working day since the pandemic, whilst family carers were asked to discuss changes to their visiting abilities in the care home. Both were asked to discuss the impact that these changes had on them. Follow-up interviews were conducted by one author (KH, a female, post-doctoral researcher) and were audio-recorded, with verbal consent obtained and recorded at the beginning of each interview. Follow-up interviews explored changed experiences since baseline, including visiting abilities in light of new public health restrictions. Interviews lasted 29(+/− 11) minutes at baseline, and 24(+/− 7) minutes at follow-up.

### Data analysis

Interviews were transcribed, anonymised, and each blindly coded by two members of the research team (KH, CG, SM, JC and MG) and one assistant psychologist, who are all experienced in qualitative analysis. Given the breadth of (often emotional) information emerging from the interviews, and the varied backgrounds of the research team members, double coding the transcripts was deemed necessary to ensure all key topics were identified for discussion, in order to allow for conceptualisation of themes. During research meetings, conversation was natural and in depth, as multiple members of the research team were familiar with each interview. The research team members were also involved in the data analysis of the baseline interviews, and so were able to compare findings from the baseline and follow up interviews easily, over multiple group discussions. As separate topic guides were used for the care workers and family carers, interviews with each group were analysed and discussed separately, before combining in the final analysis. Participants did not check transcripts for accuracy, as these were professionally transcribed for accuracy, and public involvement was integral to all stages of work, ensuring findings were relevant. Data were analysed using descriptive, inductive thematic analysis [21], and the final, conceptualised themes were discussed with carers to ensure mutual agreement.

In terms of reflexivity, the authors have a varied range of professional backgrounds (academic researchers, medicine, allied health care, social care, primary care, psychology and previous care experience). All members of the research team have experience of qualitative research, and have contributed their differing perspectives in research discussions to agree the overall findings. As one author conducted the follow-up interviews, the team-based reflection was crucial in strengthening the development of the study documents, and the data analysis and interpretation.

### Public involvement

One current and two former unpaid carers were involved in all aspects of the study, including study document design, contribution to group discussion, and interpretation and dissemination of findings. Public involvement fees were paid according to NIHR INVOLVE (2005) guidelines.

## Results

### Demographics

A total of *n* = 42 participants (26 family carers and 16 care home staff) were included (see Table 1). Of these, 11 family carers and nine care home staff participated in a follow-up interview. Of the 26 family carers, five reported that their relative/PLWD died in the care home during the time of COVID-19. The remaining 21 family carers spoke of their communication with care homes around possible EoL care during the pandemic, and how this affected them emotionally, whilst care home staff reported the new EoL care protocols in place in their care home.

**Table 1:** Demographics of included participants

	Family carers baseline (*n* = 26)	Family carers follow up (***n*** = 11)	Care home staff baseline (*n* = 16)	Care home staff follow up (***n*** = 9)	Total sample (n = 42)
	***N*** (%)	***N*** (%)	***N*** (%)	***N*** (%)	***N*** (%)
Gender					
Female	18 (69.2%)	8 (72.7%)	13 (81.3%)	8 (88.9%)	31 (73.8%)
Male	8 (30.8%)	3 (27.3%)	3 (18.8%)	1 (11.1%)	11 (26.3%)
Ethnicity					
White British	22 (84.6%)	10 (90.9%)	13 (81.3%)	7 (77.8%)	35 (56.5%)
White Other	2 (7.7%)	1 (9.1%)	1 (6.3%)	1 (11.1%)	3 (4.8%)
BAME	2 (7.7%)	0	1 (6.3%)	0	3 (4.8%)
Prefer not to say	0	0	1 (6.3%)	1 (11.1%)	1 (1.6%)
Relationship with PLWD			–	–	–
Spouse	9 (34.6%)	3 (27.3%)			
Partner	1 (3.8%)	0			
Adult child	16 (61.5)	8 (72.7%)			
Dementia subtype			–	–	–
Alzheimer’s disease	8 (30.8%)	4 (36.4%0			
Mixed dementia	2 (7.7%)	0			
Vascular dementia	4 (15.4%)	2 (18.2%)			
Lewy Body dementia	6 (23.1%)	4 (36.4%)			
Other	2 (7.7%)	1 (9.1%)			
Unknown	4 (15.4%)	0			
IMD Quintile^b^					
1 (least disadvantaged)	11 (42.3%)	6 (66.7%)	3 (23.1%)	2 (28.6%)	14 (43.8%)
2	4 (14.5%)	2 (22.2%)	3 (23.1%)	2 (28.6%)	2 (21.9%)
3	0	0	3 (23.1%)	2 (28.6%)	3 (9.4%)
4	3 (11.5%)	0	1 (7.7%)	0	4 (12.5%)
5 (most disadvantaged)	1 (3.8%)	1 (11.1%)	3 (23.1%)	1 (14.3%)	4 (12.5%)
Job role	–	–			–
Activity Coordinator			1 (6.3%)	0	
Care home liaison			1 (6.3%)	1 (11.1%)	
Care quality			1 (6.3%)	0	
Care assistant			4 (25.0%)	3 (33.3%)	
Senior care assistant			2 (12.5)	0	
Night care assistant			1 (6.3%)	1 (11.1%)	
Housekeeper			1 (6.3%)	1 (11.1%)	
Matron			1 (6.3%)	0	
Manager			4 (25.0%)	3 (33.3%)	
	**Mean (SD), [Range]**				
Age^a^	62.3 (±9.5) [42-89]	61.1 (±5.2) [51-68]	41.8 (±16.6) [18-62]	43.3 (±17.2) [21-60]	54.8 (±15.9) [18-89]
Years of education	17.9 (±2.9) [11-23]	18.09 (±1.5) [16-20]	15.7 (±2.7) [11-20]	16.4 (±2.6) [11-19]	17.1 (±3.0) [11-23]
Care home capacity	41.5 (±17.4) [18-76]	38.9 (±18.2) [18-76]	42.2 (±15.8) [12-64]	49.7 (±11.6) [36-64]	41.7 (±16.6) [12-76]
Years working in a care home	–	–	9.3 (±10.6) [1-35]	7.0 (±11.1) [1-35]	–
Years since dementia diagnosis	6.7 (±3.6) [2-16]	7.0 (±4.4) [2-16]	–	–	–
Years (PLWD) residing in a care home	2.7 (±2.1) [1-10]	2.8 (±1.9) [1-7]	–	–	–

^a^n = 1 care home staff = prefer not to say, ^b^n = 4missing data

### Thematic analysis

Three central themes were conceptualised, and redefined through research team discussions (see Table 2).

**Table 2:** Themes and subthemes following thematic analysis

Theme	Subtheme
1. Wasting or losing time	• Fear of losing touch • Breaking visiting promises, and feeling abandoned in the final days of life
2. Maintaining control, plans and routine	• Disrupted care plans and loss of control/autonomy • Exceptional visiting abilities or contact with residents • The key role of care workers, and the importance of good communication
3. Coping with loss, and a lack of support	• Lack of support for families during EoL care • Lack of support offered to those left behind

### Wasting or losing time

Theme 1, Wasting or losing time, describes the sense of urgency noted in caring for a person at EoL, where visiting was deemed immediately necessary as the resident’s life expectancy was poor. The subthemes (Fear of losing touch, and Breaking visiting promises, and feeling abandoned in the final days of life) within theme 1, highlight the fear that emerged, whereby residents may forget their relatives in the time without visits, due to their health, or would feel abandoned by their relative in cases where they have not forgotten their relatives, but have forgotten the circumstances of the pandemic.

#### Fear of losing touch

As a result of lockdown restrictions, visitors were prohibited from entering the care home, and later offered video calls or adapted visits in pods or at windows. However, for those residents in the last month/weeks of life, adapted visits were often unsuitable due to their poor health.

Some of the residents are end of life so they can’t get out of bed, so they're the limited ones who can’t really see their families unless it’s through a window which isn’t the same… ID04, female housekeeper

Family carers described a sense of urgency in seeing their relative, with a fear that they might not be able to again. These fears were heightened by the general stress of the pandemic, the media coverage about the high rates of COVID-19 in care homes, and from reports of COVID-related deaths in their relative’s home. Thus, families further worried that their relative may contract COVID-19 during the time when visiting was disallowed.

Back in April/May they [the care home] actually lost…a third of the residents, the problem was I didn’t know until it was in the newspaper…I was rather horrified ID01, female carer, spouse

The progressive nature of dementia added further strain on those family carers unable to visit their relatives, with fears that the resident may deteriorate during this time and not remember them when visiting is finally allowed. Therefore, the prospect of residents no longer remembering their relatives before the EoL was an added worry for the families.

I understand why we want to try to keep this dreadful illness out of care homes…but certainly 95, 98% of [residents], we know they’re only going to leave there at the end of their life. I think possibly we could do a little bit more to try to improve the quality of life...I’m not sure we’re giving that quite enough emphasis ID22 male carer, partner

Care home staff observed deterioration in the residents’ health and wellbeing, which they assigned to the lack of visiting and contact with relatives, validating families’ concerns. Both family and staff observed residents becoming more reliant on the care home staff, which increased families’ anxieties that they will become forgotten. Thus, the lack of visiting negatively impacted the residents, staff and families separately. The families’ grief and distress were further exacerbated up to the point of bereavement, fearing that residents would die without recollection of being loved and cared for.

It’s really hard, it’s almost like she [PLWD] relies on the staff now which is nice in one way…but it’s like she [gets] upset and hugs them [pause] as soon as mum went into the home she deteriorated terribly within 2 weeks…But since the corona…I just feel she’s missing… that connection ID11, female carer, spouse

#### Breaking visiting promises, and feeling abandoned in the final days of life

Families described initial feelings of guilt when the time came for their relative to enter a care home, of which they struggled come to terms with, often forming new routines, frequently visiting the care home, as a means of coping with this change. Additional fears that relatives would feel abandoned in their final days of life were prominent amongst interviews.

Going into a care home, she probably…thought that’s it, they’re just going to dump me, I’m not going to see anyone again, so I do wonder whether during COVID that’s what she thought and I did wonder when she went into hospital has she just given up and just thought “I’ve been abandoned and dumped” ID03, female carer, daughter

During the pandemic, however, family worried that their relative would feel confused and abandoned without frequent visiting. Where residents entered a care home at the beginning of/during lockdown, family carers were forced for break previous visiting promises due to the lockdown restrictions, and feared the residents would not survive the pandemic with these final feelings of abandonment.

I promised [PLWD]…that there would never be two consecutive days where she didn’t see me… It [lockdown] was all very sudden…obviously I did my best to explain to [PLWD] that I would see her again as soon as I could but I couldn’t put a date on it. And obviously I felt really guilty then because it meant I was breaking my promise ID22, male carer, partner

Care home staff furthered this notion, with reports that residents with dementia did not understand the visiting rules, and were often confused as to why their family had stopped visiting them, with staff reporting similar feelings of guilt due to the impact this caused on the residents.

They do ask where their family is…some of the residents think that it’s us not letting their family in, they can be quite upset about that until we explain it to them, but it’s hard isn’t it, I mean it’s hard for anyone to grasp ID09, female senior care assistant

### Maintaining control, plans and routine

Theme 2, Maintaining control, plans and routine, illustrates participant accounts of attempting to control some of the adverse circumstances brought on by the pandemic, in caring for a resident at EoL. The subtheme, Disrupted care plans and loss of control/autonomy, describes the detailed care planning that families’ put in place prior to the pandemic, in order to best care for, and prepare for the loss of, a relative in a care home. However, during the pandemic, family described a loss of autonomy in EoL care planning. Further subthemes (Exceptional visiting abilities or contact with residents, and The key role of care workers, and the importance of good communication) portray the key role of the care home staff during this time, who mediated contact between families and residents during the pandemic restrictions. The importance of clear and truthful, communication from the care home staff, and their role in facilitating visits, are described in this theme, with a lack of supporting guidance highlighted for care home staff.

#### Disrupted care plans and loss of control/autonomy

Family carers described careful planning that took place prior to the pandemic, which often included preparation for EoL. Decisions to attain power of attorney, frequent visits and maintaining an active caring role, allowed families to retain control that was otherwise lost when their relative began residing in a care home. Therefore, EoL care planning attempted to neutralise power imbalances between institutional and family caregiving, allowing residents and/or families to choose where and how to die, often in a natural setting with family surrounding them. However, during the time of COVID-19, families experienced a significant disruption to the previous care plans, and a loss of control, which added to feelings of stress.

In an end of life situation… you’re in full PPE and you’ve got to keep two meters away…I said well that would be like me going and watching my mother dying. I wouldn’t be easing her, I wouldn’t be comforting her, I’d just be sat watching, that to me is quite horrific really and…I think I’d want my sister with me obviously…she’s an equal daughter, isn’t she? ID14, female carer, daughter

The residents reportedly have a General Practitioner who attends the care home regularly, and who were integral in the care decisions made throughout the pandemic. However, family carers reported shock and distress when approached by the healthcare provider, via a letter in the post, to sign a “Do Not Resuscitate” (DNR) form (without appropriate advice or guidance) on behalf of their relative in a care home, to avoid hospital admittance for medical treatment in the event of illness during the pandemic due to a lack of beds in intensive care units. Families were shocked as their relative in the care home was deemed relatively young and healthy despite living with dementia. Their refusal to sign indicated both a breakdown in communication regarding such a sensitive topic, and an attempt to regain control over their relative’s care.

The doctor wrote to everybody, all the relatives of the residents… saying that…if hospitals become overwhelmed …elderly patients and those with multiple comorbidities will most likely be assessed as low priority for intensive care beds [pause] my wife is frail… I wouldn’t call her elderly…she might not be considered for treatment [pause]…and in the end I didn’t say I would agree to a DNR order under the current situation ID07, male carer, spouse

#### Exceptional visiting abilities or contact with residents

Both positive and negative experiences of visiting a relative at EoL care emerged from narratives of family carers. Those who felt the EoL care was conducted well, allowing them to spend meaningful time with their relative at the end, acknowledged that the visiting abilities offered by the care home likely breached restrictive COVID-19 measures. These discussions were supported by the interviews with care staff, who further reported using necessary means of getting around the guidance in order to support contact visits. Overall, these measures clearly supported the family carer to prepare and grieve the loss of their relative, in fitting with their pre-COVID19 care planning.

They [care staff] said to me…why don’t you basically move in… I cannot praise the staff highly enough… from that point I spent about 22, 23 hours a day at the home…they fed me, they made sure I was comfortable…they even said look get into bed with her if you want. ID22, male carer, partner

We have an essential visitor status and it was quite open…for interpretation…we increased it because…we could see that [residents] were failing…and I think we had about 50% of ours [visitors] even more than that probably able to come in and spend some times with their loved one ID06, female

Family carers, who were not offered exceptional visiting, viewed the EoL care plan as inhumane and insensitive, adding to previous feelings of fear and worry. These participants were informed that only one family member could enter the home when EoL was suspected, and no physical contact would be allowed, for risk of the virus spreading through the home.

What they [care home staff] were saying was…say I sat really close to my mum and I was touching her, breathing on her whatever and mum caught COVID from me, even though she was dying, and then a nurse came in to speak to her, then that’s how it could spread through the home. But then I was thinking surely the nurse would have her mask on and it just seems a bit overkill to me in a very sensitive situation ID14, female carer, daughter

#### The key role of care workers, and the importance of good communication

With the absence of contact visits, it was suggested that residents formed closer relationships with the care home staff, causing mixed-emotions for the family. Despite feeling detached from their relative’s care, the family were reassured in knowing that their relative was content and well looked after. The family further empathised with the staff due to the extra burden placed on them, particularly in supporting residents on EoL care, as this number quickly multiplied in the early stages on the pandemic.

He [PLWD] was very close to some of the carers actually…he made a big mark on their hearts…you could just tell that they had made that real connection with my dad and they would be saying that he talks about you all the time…so I think [that helped because] we worried that he might have felt abandoned ID15, female carer, daughter

In addition, family became increasingly reliant on the care home staff communicating information regarding the resident’s wellbeing. Where communication was clear and consistent, family were grateful for the extra efforts made by the staff to remotely involve them in their relatives’ care.

They [care staff] were really good, they didn’t hide things…and when I phoned up, they’d update me and stuff and let me know how she [PLWD] was and they were very honest about it ID03, female carer, daughter

However, communication was not always consistent amongst participants, resulting in increased stress and confusion, particularly when a relative was deemed to be EoL. Care staff reported that constantly changing government guidance made it difficult to provide clear information to families, and began to lose the families’ trust and support over time.

We were absolutely bombarded in the beginning with different guidance and I suppose that’s because people were learning from experience of COVID…the guidance was regularly changing and it was getting confusing to the point where even families were thinking we were making up guidance

ID18, male care home manager

### Coping with loss, and a lack of support

The final theme, coping with loss and lack of support, describes the emotive accounts of those left behind following the loss of residents during the pandemic. The subthemes (Lack of support for families during EoL care, and Lack of support offered to those left behind) discuss the lack of support offered to both family and staff caring for residents at the EoL, and following their death. Participants spoke of grief and stress during the pandemic, however, some expressed accounts of good care and gratitude for the time and contact they had with their relative/PLWD at the EoL.

#### Lack of support for families during EoL care

During the pandemic, family carers were affected from the loss of not being able to visit their relative, and from a lack of communication from the care home. Families described feeling helpless and detached from their previous caring role, and expressed a desire to be involved in the practical discussions and decision making up to, and at the point of, EoL.

We had to still be apart so she couldn’t touch me and sense me and it was always actually a very you know touching relationship, so, it did mean that we were missing out on a fairly important part of our relationship ID22, male carer, partner

It was felt that the adapted visiting options, when possible, did not provide the meaningful contact that family need at EoL. This resulted in further, negative impacts to the family carers’ emotional wellbeing, during an already distressing period.

It would have been nice maybe to have a kind of Skype with a key worker, once a week or even once a fortnight to kind of say this is how you know your dad’s been, this is what we’ve done, this is how his care plan’s changed but we there was nothing like that ID15, female carer, daughter

#### Lack of support offered to those left behind

The family carers whose relative died during the pandemic expressed a general lack of closure due to the lack of meaningful contact, with many only allowed to visit when the resident was dying and/or via a video call. Furthermore, the UK government’s restrictions on funerals and large gatherings of people at that time further limited the families’ abilities to grieve, or to benefit from family support in coming to terms with the loss.

My dad’s funeral for example… with the thing of different households and… as a family, having to keep your distance…when you’re not [in] the same household… I think it does affect me… I think that’s where most of my pandemic challenge comes from on an emotional level ID15, female carer, daughter

Moreover, care home staff furthered feelings of loss and a lack of support in coping with increased EoL care and subsequent deaths in care homes during the time of COVID-19. Staff reported difficulty in coping with these emotions whilst also contending with the pandemic restrictions in their personal life.

It’s hard to hear that somebody that you care for died every day for two weeks…it takes a big impact, that was really tough and then with the lockdown restrictions I just think everyone [care home staff] was feeling it as well ID04, female housekeeper

Therefore, it was clear that both care home staff and family carers suffered loss during the time of the pandemic, without sufficient support during or after the loss of residents.

## Discussion

As care homes were not prioritised, particularly in the early stages, of the pandemic, support structures for residents, their families and care home staff were left to individual development. As a result, service provision varied significantly and impacted care experience at the EoL. The evidence reported here is of the first to highlight the effect that variable EoL care provision had on those working in the care home with dementia residents, and especially on the family carers unable to be with their relatives at the EoL, and how this impeded their ability to cope with the bereavement.

A significant finding from our research describes how some families were informed that only one family member could visit the home at EoL, and no physical contact would be allowed. However, pre-pandemic national guidance for supporting EoL care, recommends individualised care, including personal goals and wishes, communication and shared decision making are provided, contradicting the present restrictions [22]. The Marie Curie “Better End of Life” report reiterates that people should be given the chance to choose (where possible) to spend their EoL, with the appropriate support and care [7], but notes that there is “inequity” in achieving this [7]. Health inequity refers to unfair and avoidable differences in health between different groups of people [23], and inequitable care exists where good EoL care remains a “postcode lottery ”[7]. The current study evidence adds to these findings, whereby some relatives reported meaningful contact at EoL (albeit in breach of COVID-19 guidance), and others offered only a video call with their dying relative. Video calls, window visits, or a contact visit with only one family member, may offer some comfort to care home residents and their families, but was deemed insufficient when EoL was suspected or imminent. Despite the risk that the COVID-19 virus poses to care home residents and workers, families’ have highlighted an immediate need for contact with residents at EoL, more so than those who are not at EoL. Without regular visits, families reported fears that residents would forget them before the point of death, or would forget the circumstances of the pandemic leading to feelings of abandonment before death. It is therefore, a recommendation from this study that national guidance supports a minimum level of safe contact with relatives at EoL, offered equally to family members, to support their grieving and avoid subsequent negative impacts to their emotional wellbeing. As national EoL care guidance remains unattainable during the pandemic, robust structures, careful planning and improved communication, must be put in pace to facilitate the following of this guidance. However, the findings from this research specially relate to participants’ experiences of caring for residents within care homes, and it should be noted that differing guidance around equitable EoL visitation may be required for those in alternative care settings.

Advance care planning is vital in ensuring equitable care, especially in cases of dementia where rapid deterioration may prevent consultation with the PLWD [24]. Despite the prior efforts made to meticulously plan for a relative entering into a care home, and their subsequent EoL care, family carers reported a loss of control, and exclusion from their relative’s care following visiting restrictions. Previous routines and care plans faltered or were lost entirely due to the disruptions of the pandemic. Furthermore, family carers were shocked when presented with DNR forms with little/no guidance and information; a conversation that would previously have been included in EoL discussions between healthcare providers and the family [25]. However, if signed, these forms would indicate that their relative would not be hospitalised for intensive care, a notable difference from the use of DNR forms pre-pandemic, that solely indicated DNR in the event of fatal illness. Evidence has emerged portraying an ethical dilemma in the application of DNR discussions during the COVID-19 pandemic [26]. Stretched hospital services in the peak of the pandemic were forced to evaluate resource allocation carefully, namely the need of a ventilator for successfully resuscitated patients [26, 27]. It is clear from the current evidence that the lack of communication accompanying such a request was a significant oversight, causing concern in an already distressed group.

Recent qualitative research reported that health and social care professionals were central in creating contact between patients with their families at EoL during the pandemic [28]. However, examples of communication breakdown, especially at the EoL when families are desperate for information, were evidenced from the current study, whilst the care staff reported inabilities to meet these needs based on frequently changing guidance. It is suggested that by offering better support to care home staff, they can be alleviated of increased work demands, allowing for better communication and facilitation of visits [28]. However, based on the current study findings, a further suggestion should be made for consistent guidance, especially for EoL care, allowing care staff to share clear and trusted information to families.

A lack of support was reported by family carers during and following the death of a care home resident, impacting their ability to grieve and cope with their loss. It has been previously identified that family caregivers begin the bereavement process in a state of emotional and physical exhaustion from the caregiving experience [12, 29]. However, the current study findings add to this, through reporting further impacts caused by insufficient contact visits before the point of EoL (where PLWD can still engage in meaningful interaction with their relatives). Furthermore, the inability to gather and hold funerals due to lockdown restrictions indicates that today’s family carers experience further barriers to coping with loss, highlighting a greater need for support to be offered to relatives in coming to terms with the impending, and long-term, loss of a care home resident.

Reports showed that bereaved relatives during the pandemic unable to visit their relative at the EoL, could not be adhere to lockdown restrictions under the circumstances [13, 14]. However, the current findings identified that care home staff also reported similar experiences, where they felt unable to restrict family visiting, and even turned a blind eye to contact visits, when it was felt that EoL was imminent. In addition, the current study identified the impact of restricting visits between families and residents living with dementia, as time was deemed to be wasted each day they could not visit, and family feared their relative would no longer remember them at the EoL. Therefore, it can be inferred that the pandemic guidance on restricting visits in care homes was unrealistic and did not meet the needs of the residents, families or the staff delivering EoL care, indicating a need for change and consideration in the current, and possible future, pandemics.

Based on the current study data, and linked with developing data [14, 28], the following recommendations are drawn:

1. EoL care should be consistent between care homes and between residents. Therefore, clear guidance must be provided to care homes in order to ensure all are providing the same level of equitable, safe care nationally.
2. Communication between care home staff and residents’ families should be organised, consistent and frequent, to ensure families remain an active part of their resident’s care, and are involved in important decision making.
3. Clear guidance and communication should be provided to families regarding the signing of DNR forms.

## Limitations

BAME participants were underrepresented due to the convenience sampling method. The current study is unable to report information on UK region, although the authors acknowledge that this was a variable factor to consider due to the differing restrictions across the country at that time. Information on area of residence and place of work cannot be shared in this research to protect the anonymity of the participants. In addition, although the staff participants provided insight into their experiences of delivering EoL, and family carers spoke of their fears of encountering EoL care during the pandemic, only five participants had specific experience of a relative dying in a care home during the pandemic. Future research should consider alternative recruitment strategies to better capture the views of a broader population.

## Conclusion

Adapted care home visits during the pandemic were deemed insufficient when EoL was suspected or imminent. Lack of suitable, meaningful visits with PLWD in care homes resulted in negative feelings abandonment from both family and care staff, with fears that the PLWD may forget their relatives without frequent visiting prior to EoL. Where families experienced positive EoL visits, these were in breach of public health restrictions at that time. It is therefore, recommended that care homes receive clear guidance offering equitable contact with relatives at EoL to all family members, to support their grieving in the short and longer-term.

## Supplementary Information

**Additional file 1: ** Topic guides for use with family carers, and care home staff.

## Data Availability

The data that support the findings of this study are available on request from the author [CG]. The data are not publicly available due to ethical restrictions.
