# Supplementary material for: End of life care in UK care homes during the COVID-19 pandemic: a qualitative study
Source: BMC Palliat Care. 2022 Jun 1;21:91. doi: 10.1186/s12904-022-00979-4 (PMC9155982; doi:10.1186/s12904-022-00979-4)
Supplement: Supplementary file 1 — Additional file 1: Topic guides for use with family carers, and care home staff. [file 12904_2022_979_MOESM1_ESM.docx]

**Appendix 1**

**COVID19 Care home study topic guide – Follow-up interviews**

**- Family carers -**

Since we last spoke to you in autumn last year, have there been any changes to your caring situation, or your relative’s wellbeing?

Any changes to your relative’s health and wellbeing?

Any changes to your own health and wellbeing?

Since we last spoke to you what, if any, changes have there been in visitation to the care home where your relative resides?

And have you been able to visit your relative at the care home since autumn?

If yes - how were these visits undertaken?

How did you feel about the visits – were they positive or negative?

Have you or your relative been vaccinated yet?

If yes, what, if any, difference has this made to visiting permissions and arrangements to see your relative?

If yes, when did you/your relative get the vaccine, and how did it make you feel when it was announced that this was happening/how does it make you feel now knowing that you/relative have been vaccinated?

If no, do you know when you/your relative might be vaccinated? And what do you think about this – will you accept a vaccine (and why)?

What about the communication, advice and support you have been getting from the care home more generally? And what about government communication and guidance?

Can you tell us more about how the care home has updated relatives on the vaccination?

Moving forward (post vaccine), how do you envisage the visitations rights to change, bearing in mind vaccination does not give full protection?

What about the future- how do you predict things may change looking forward into 2021 and beyond?

Finally, is there anything else you feel you wish to share that we haven’t covered yet?

**Appendix 2**

**COVID19 Care home study topic guide – Follow-up interviews**

**- Care home staff -**

How have you been since we last spoke around last autumn 2020, - have there been any changes in how you are delivering care at the care home?

Since last autumn, has your role changed or are you still in the same role?

Have you been vaccinated? If so, when was this? If not, why not?

How has the uptake of vaccination been at your care home for both staff and residents?

Who delivered the vaccines?

What were your experiences surrounding the vaccination process?

Have you faced any further difficulties in delivering care during the pandemic? PROMPT: Access to PPE, testing, vaccination

Have staff been able to access any (new) support?

How have family visits been taking place since we last spoke? Have testing and/or vaccination supported safe visitation?

Has communication from the government surrounding care home visitation advice changed and has it been consistent?

How do you now feel about the future of care homes and especially visitation of family members?

And finally, any other key points we’ve missed that you’d like to tell us about?
